# Selective Visual Attention during Mirror Exposure in Anorexia and Bulimia Nervosa

**DOI:** 10.1371/journal.pone.0145886

**Published:** 2015-12-29

**Authors:** Brunna Tuschen-Caffier, Caroline Bender, Detlef Caffier, Katharina Klenner, Karsten Braks, Jennifer Svaldi

**Affiliations:** 1 Department of Psychology, University of Freiburg, Freiburg, Germany; 2 Klinik am Korso, Bad Oeynhausen, Germany; 3 Department of Psychology, University of Tübingen, Tübingen, Germany; Charité-Universitätsmedizin Berlin, Campus Benjamin Franklin, GERMANY

## Abstract

**Objective:**

Cognitive theories suggest that body dissatisfaction results from the activation of maladaptive appearance schemata, which guide mental processes such as selective attention to shape and weight-related information. In line with this, the present study hypothesized that patients with anorexia nervosa (AN) and bulimia nervosa (BN) are characterized by increased visual attention for the most dissatisfying/ugly body part compared to their most satisfying/beautiful body part, while a more balanced viewing pattern was expected for controls without eating disorders (CG).

**Method:**

Eye movements were recorded in a group of patients with AN (*n* = 16), BN (*n* = 16) and a CG (*n* = 16) in an ecologically valid setting, i.e., during a 3-min mirror exposure.

**Results:**

Evidence was found that patients with AN and BN display longer and more frequent gazes towards the most dissatisfying relative to the most satisfying and towards their most ugly compared to their most beautiful body parts, whereas the CG showed a more balanced gaze pattern.

**Discussion:**

The results converge with theoretical models that emphasize the role of information processing in the maintenance of body dissatisfaction. Given the etiological importance of body dissatisfaction in the development of eating disorders, future studies should focus on the modification of the reported patterns.

## Introduction

Anorexia nervosa (AN) and bulimia nervosa (BN) are characterized by eating behavior disturbances and cognitive-affective disturbances regarding the body and the self. Body image disturbances include a wide range of perceptual, cognitive-affective and behavioral phenomena [1–5]. As shown in meta-analyses and reviews [6, 7] perceptual phenomena are less specific and pronounced for individuals with eating disorders than cognitive-affective phenomena, whereby individuals with BN self-report higher levels of cognitive-affective body image disturbances than persons with AN [6].

Body image disturbances, especially cognitive-affective ones, do not only accompany, but in conjunction with behavioral risk factors such as dieting also predict the development of eating disorders [8–11]. Furthermore, they are vital factors for maintenance [12] and treatment outcome of eating disorders [13, 14]. Body image disturbances can be explained as results of dysfunctional schemata regarding the self and the body, which influence and distort a variety of mental processes [15]. In the course of information processing, attentional processes play an important role, as they interfere at an early stage und thus determine following interpretation, evaluation and their consequences (i.e., experiences). In the context of eating disorders, distorted and biased attentional processes presumably maintain body image disturbances by narrowing and (re-)directing the scope of information being processed to schema-consistent contents, while schema-inconsistent contents are not attended to and are thus neglected. Studies of attentional biases in eating disorders indeed have shown that eating disorder psychopathology is linked with vigilance to such stimuli, resulting in impaired or enhanced performance in attentional tasks [15–18]. However, results have not always been consistent and experimental paradigms employed in these studies, such as the modified stroop task, dichotic listening, lexical decision tasks or dot probe tasks have been criticized for methodological and interpretation ambiguity [16, 19–21].

In order to decrease interpretation ambiguity and increase ecological validity of stimuli, studies in the last decade have utilized eye tracking devices to assess attentional processes during the exposure to salient stimuli, e.g., the body. In the non-clinical domain, one study [22] found that high body dissatisfied women locate their gazes longer and more frequently to thin bodies rather than towards other types of bodies, compared to women with low body dissatisfaction. In another study [23] women with high body dissatisfaction were found to allocate their attention significantly more often and longer towards hips, waist, legs and arms than women with low body dissatisfaction do. By contrast, in another study [24] women with a high compared to those with a low level of drive for thinness tended to avoid body parts often indexed as problematic, as displayed by a shorter gaze duration towards the respective body zones. Other studies have found similar avoidance behavior [25].

Given these contradictory results, recent studies focused on the time course of the attentional processing of body-related cues [26, 27]. In one of these studies [27], dissatisfied compared to satisfied women displayed an initial orienting, speeded detection and increased maintenance bias towards pictures denoting fat physics relative to neutral pictures. Furthermore, dissatisfied compared to satisfied women were characterized by a speeded detection for thin body pictures compared to neutral pictures. In the other study [26] dissatisfied women were characterized by a sustained maintenance bias on thin and fat body images during early and later stages of the information processing stream. Thus, biased attention towards body images seems to be driven by both bottom-up and top-down processes and these processes may also be active with regard to the attention allocation towards specific body parts.

In the domain of AN and BN only a few studies have tested visual processing of thin and fat body pictures. Thereby, one study [28] found both females with an eating disorder (AN or BN) and healthy controls to preferentially process body parts often indexed as unattractive (e.g., hips, upper legs) relative to other body parts. In this study though only girls with AN and BN displayed an attentional bias towards unclothed body parts. The authors interpret this as the behavioral expression of the overvaluation of shape and weight manifested in self-report measures of body dissatisfaction and drive for thinness. In another study [29] females with AN compared to healthy controls gazed longer towards images of thin and fat bodies relative to images of social interactions, but displayed the strongest attentional bias for images of thin bodies. Taken together, several studies yield evidence of a differential attentional processing of images of thin and fat bodies, however, research in this domain is inconsistent, which–among others- may be due to the developmental stage of participants (adolescence vs. adulthood), stimulus material (fully, partially dressed) and the duration of stimulus presentation.

Furthermore, several other studies investigated whether eating disordered individuals process their own and other bodies in fundamentally different ways. As such, one study [30] assessed visual attention towards the own body relative to a photo of a matched control participant’s body in women with AN, BN and healthy controls by means of a modified dot-probe paradigm. Saccade latency was used as an index of covert attention to the cue photos. In the AN group saccades were faster when the self-photo was the target whereas in the BN group there was a numerically opposite but non-significant pattern. Control participants displayed an even attention allocation towards the self and other-photo. Comparably to women with AN, women with BED were shown to display an increased attention towards the self relative to the other photo compared to weight-matched controls without BED [31]. These results evidence, that different mechanisms of body dissatisfaction maintenance may be active within the various eating disorder groups, and the question arises, whether such differences occur also with regard to the attentional distribution within one’s own or the body of another person.

As such, extending previous work [32], one study [33] found that women with eating disorder symptoms spend more time looking at their least liked than most like body parts whereas they spent more time looking at most liked than least liked body parts in others. Woman without eating disorder symptoms showed the reverse pattern. Thus, eating disorder symptoms appear to be accompanied by a lack of body-related self-serving bias [34] and higher vigilance for schema-consistent information. In a similar vein, women with higher BMI and lower levels of self-rated attractiveness where shown to be characterized by an increased attention allocation towards their most unattractive and an increased attention towards the most attractive part of the control body [35]. By contrast, another study [36] found women with AN compared to healthy controls to display a shorter gaze duration on (self and others’) body parts they have a particularly negative perception of (breasts and thighs). This however is indicative of an avoidance, rather than a hypervigilance towards negatively evaluated body parts and contradicts previous results [33, 35, 37]. Among others, these contradicting results may be due to the nature of the visual assessment, in that some instructions and settings may more or less enable avoidance behavior during a free viewing task.

The present study aimed to assess the attentional distribution with regard to the most valent body parts in women with AN and BN. Instead of using a photo of the participants, eye-movements were recorded during a body exposure in front of a mirror to increase ecological validity. To better control for avoidance during the assessment of visual patterns, participants’ eye movements were assessed using a think-aloud procedure. In addition, we included beauty and satisfaction ratings in order to control for differential effects. Corresponding with previous studies, [33, 35], women with AN and BN were expected to display longer and more frequent gazes towards their most ugly relative to their most beautiful body part. The same pattern was expected for their most and least satisfying body part. For participants in the control group, a more balanced distribution of eye movements was expected.

Furthermore, on the background of the reported higher levels of cognitive-affective body image disturbances in women with BN compared to AN [6], gazes of higher duration and frequency towards the most ugly/the least satisfying body part were expected in women with BN compared to participants with AN and healthy controls. Finally, during the exposure the eating disorder groups were expected to experience more discomfort and to self-report a higher arousal than the healthy control group [38–40].

## Materials and Methods

### Participants

Sixteen female inpatients with AN, 16 with BN and 16 female participants without lifetime eating disorder (control group; CG) participated in this study. The study was approved by the ethics committee of the University of Bielefeld (EUB 2015–097). Written informed consent was obtained from all participants.

The eating disorder groups were patients of an inpatient clinic specialized on eating disorders. All patients participated in the study before their treatment program started. Patients were required to meet criteria for AN or BN based on the DSM-IV [41], applying the German version of the Structured Clinical Interview (SCID) for DSM-IV [42, 43] and the German version of the Eating Disorder Examination Interview (EDE, [44, 45]). Exclusion criteria for both the control group (CG) and the eating disorder groups included a body mass index (BMI) less than 14, comorbid psychotic symptoms, objections of the therapist responsible, considerable hypermetropia and age exceeding 45 years. An additional criterion for the CG was the presence of a current or lifetime eating disorder. In total, 85 patients were interested in participation and completed the diagnostic assessment session. Of those, eight (9.4%) had to be excluded due to exclusion criteria, 24 (28.2%) did not show up to the scheduled eye tacking experiment, 21 (24.7%) had to be excluded afterwards due to technical problems and missing data. Those withdrawn and excluded did not differ from participating patients in age (*F* [4, 80] = .54, *p* = .71), BMI (*F* [4, 80] = .41, *p* = .80), body dissatisfaction as measured by the Body Shape Questionnaire (BSQ) score (*F* [4, 69] = .57, *p* = .69), overall eating pathology as assessed by the Eating Disorder Examination-Questionnaire (EDE-Q) total score (*F* [4, 68] = 2.29, *p* = .07), as well as severity of depression by means of the Beck Depression Inventory (BDI) score (*F* [3, 43] = .72, *p* = .55) and the global severity index of the Symptom Checklist-revised (SCL-90-R) (*F* [4, 54] = 1.04, *p* = .40).

The CG was recruited by announcement at the local University. In total, 21 women were invited and examined with the SCID and EDE. Two participants (9.5%) were excluded due to technical problems and three (14.3%) due to missing data in the evaluation of body parts, so that the final control sample comprised 16 women. None of the participants in the CG met exclusion criteria (BMI less than 14, comorbid psychotic symptoms, considerable hypermetropia, age exceeding 45 years) or criteria for any eating disorder based on DSM-IV.

Patients with AN, with BN and the CG did not differ in age (*F* [2, 45] = 1.27, *p* = .29) or job status (*χ* ^*2*^ [4] = 7.53, *p* = .11). Expected differences however emerged on BMI, eating disorder psychopathology scales and general psychopathology scales (Table 1).

**Table 1:** Sociodemographics and psychopathology of patients with anorexia nervosa (AN), bulimia nervosa (BN) and the healthy control group (CG).

Variable	AN (*n* = 16)	BN (*n* = 16)	CG (*n* = 16)	Test statistic	*p*	Post-hoc Tests
Gender	All female	All female	All female			
Mean Age (years)	22.09 (3.29)	22.31 (6.00)	23.65 (1.34)	F(2,45) = 1.27	0.29	
Job Status (N)				χ^2^(4) = 7.53	0.11	
Unemployed	2	0	0			
Student	11	11	15			
Employed	3	5	1			
BMI	14.55 (1.15)	21.10 (2.92)	21.41 (2.80)	F(2, 45) = 40.84	< 0.001	AN < BN, CG
BSQ	115.06 (37.61)	130.81 (26.13)	57.75 (18.69)	F(2, 45) = 29.01	< 0.001	AN, BN > CG
EDE-Q_RE_	3.95 (1.48)	3.70 (1.20)	0.54 (0.52)	F(2, 45) = 44.35	< 0.001	AN, BN > CG
EDE-Q_EC_	3.93 (1.05)	3.59 (1.32)	0.31 (0.53)	F(2, 45) = 61.22	0.001	AN, BN > CG
EDE-Q_WC_	3.66 (1.41)	3.91 (1.04)	0.78 (0.66)	F(2, 45) = 41.37	0.002	AN, BN > CG
EDE-Q_SC_	4.20 (1.14)	4.63 (1.24)	0.99 (0.90)	F(2, 45) = 51.78	0.001	AN, BN > CG
EDE-Q_GS_	3.93 (0.94)	3.96 (0.98)	0.65 (0.57)	F(2, 45) = 79.53	< 0.001	AN, BN > CG
SCL_SOM_	58.73 (9.43)	58.86 (9.02)	51.13 (11.01)	F (2, 42) = 3.09	0.06	
SCL_OBC_	60.33 (9.91)	59.79 (7.37)	43.56 (10.05)	F(2, 42) = 16.41	< 0.001	AN, BN > CG
SCL_IPS_	61.53 (10.10)	63.21 (9.13)	45.56 (8.35)	F (2, 42) = 17.26	< 0.001	AN, BN > CG
SCL_DEP_	63.00 (6.47)	63.43 (8.96)	48.38 (9.95)	F(2, 42) = 15.30	< 0.001	AN, BN > CG
SCL_ANX_	57.27 (7.92)	60.50 (6.99)	47.44 (6.93)	F(2, 42) = 13.28	< 0.001	AN, BN > CG
SCL_AH_	56.20 (7.78)	56.21 (9.48)	45.50 (8.09)	F(2, 42) = 8.29	.001	AN, BN > CG
SCL_PA_	52.13 (8.43)	52.07 (10.63)	45.19 (4.87)	F(2, 42) = 3.67	0.03	
SCL_PI_	53.93 (12.33)	57.79 (9.21)	41.44 (4.27)	F(2, 42) = 13.38	< 0.001	AN, BN > CG
SCL_PS_	59.13 (9.35)	58.71 (9.91)	44.19 (7.83)	F(2, 42) = 13.77	< 0.001	AN, BN > CG
SCL_GSI_	61.20 (6.73)	61.71 (7.75)	45.44 (9.46)	F(2, 42) = 20.15	< 0.001	AN, BN > CG
BDI	25.50 (8.07)	21.63 (9.46)	3.69 (6.96)	F(2, 45) = 32.00	< 0.001	AN, BN > CG

Values in parentheses are standard deviations. Due to multiple comparisons, statistical significance was set to p = .01. T-Scores between 40 and 60 represent scores within the range of one standard deviation of the normal scores. Group differences are based on post-hoc Scheffé-tests, when F values were significant with p ≤ .01. Only significant differences are denoted. BMI = body mass index (weight/height^2^); EDE-Q = Eating Disorder Examination Questionnaire; GS = global score; RE = restraint subscale; EC = eating concerns subscale; WC = weight concerns subscale; SC = shape concerns subscale; SCL-90-R = Symptom Checklist-revised (t-scores); SOM = somatization; OBC = obsessive-compulsive; IPS = interpersonal sensitivity; DEP = depression; ANX = anxiety; AH = anger hostility; PA = phobic anxiety; PI = paranoid ideation; PS = psychoticism; GSI = global severity index; BDI = Beck Depression Inventory.

### Questionnaires and Interviews

In addition to the initial diagnostic measures (SCID, EDE), the following self-report instruments were applied: (1) The Eating Disorder Examination- Questionnaire (EDE-Q) [46, 47] is a psychometrically sound measure assessing severity of eating psychopathology [48, 49]. It comprises a total score and four subscale scores (restraint, eating concern, weight concern, shape concern). Higher scores reflect higher level of eating psychopathology. (2) The Body Shape Questionnaire (BSQ, [50, 51]) is a reliable and valid measure assessing body satisfaction. Higher scores represent higher body dissatisfaction. (3) The Symptom Checklist—revised (SCL-90-R, [52]) assesses overall psychological and psychiatric symptoms. It provides a global severity index (GSI), indicating the extent to which participants suffer from psychological-psychiatric symptoms in general in addition to several other subscales. (4) In order to control for severity of depression, the German version of the Beck Depression Inventory (BDI, [53, 54]) was administered. (5) Beauty and satisfaction of body parts were assessed using a body part rating scale, developed for the present study. Participants were asked to rate the beauty of and their satisfaction with their shoulder, belly, décolleté, thigh, arms, hip, bosom on a 6-point rating scale (1 = ugly/dissatisfied, 6 = beautiful/satisfied) after which they were asked to name the most beautiful, most ugly, most satisfying and least satisfying body part. (6) In order to assess mood and arousal at five times over the course of the experiment (see procedure), the Self-Assessment Manikin (SAM, [55]) was administered, which assesses emotional valence and arousal on a nine-point rating scale. Higher emotional valence ratings indicate more negative valence, higher arousal ratings indicate higher arousal.

### Procedure

The procedure of the present study was identical for both patients and comparison participants and was led by one of four trained psychology students. The diagnostic assessment was conducted in two appointments to reduce subjective burden and consisted of the SCID, EDE and required participants to fill out the questionnaires (despite the SAM, which was administered during the experimental session). Diagnostic sessions were supervised by the clinic staff and the first author (BTC).

In session three, the eye-tracking experiment was conducted. After familiarizing with the technical device, participants were introduced to the procedure. They were reassured that they could quit at any time without any disadvantage and they gave written informed consent. As visual patterns not automatically reflect attention allocation, we aimed to increase data validity by using the thought sampling technique, which has previously been suggested to be more sensitive for the identification of group differences than self-report cognition inventories [56]. Analogue to Hilbert and Tuschen-Caffier [57], concentration on concurrent cognitions and emotions as well as thinking aloud were trained in a standardized 20-min exercise. During this exercise, participants rested in a comfortable chair, were asked to relax and to allocate their attention towards what was going through their mind at the very moment. They were then instructed to speak aloud everything coming to their mind, while they were looking at some pictures.

Participants then completed the SAM scales (time 1). Following a 5-minute period of guided relaxation, they completed the SAM once more (time 2). Then they changed into standardised underwear (cream panty and cream top) and were asked to remove make up. They were then instructed to stand in front of a closed mirror (distance approx. 1.5 meters) behind a shoulder-high paravent, while the experimenter accommodated and calibrated the eye-tracking device. The mirror was opened for participants to adapt to the look of their head and the eye-tracking device in the mirror. Because of the paravent, they could not yet see their bodies. When calibration was completed, the experimenter went out of sight, the eye tracking recording device was started and the paravent removed. Participants were then instructed to look at the mirror and to think aloud again for three minutes, while their eye movements were recorded. Then, the eye-tracking device was removed and participants filled the SAM once more (time 3). Having dressed, they filled the SAM again (time 4).

The experimenter conducted a post-experimental interview, during which participants were emotionally stabilized and informed about the objectives of the study. Finally, a guided relaxation routine was offered (alternatively reading a book) for 25 minutes. After that, participants completed the SAM for the last time (time 5).

### Data preprocessing

The eye-tracking recording consisted of 25 pictures per second and was analyzed using Interact 7.1.x (Mangold GmbH, Arnstorf). For each picture, the experimenters identified the body part on which the cursor was set (representing the fixation direction of the eye). Individually, duration in sec (i.e. sum of pictures coded for this body part divided by 25) and frequency (i.e. number of sequences of consecutive pictures coded for the same body part divided by the number of all such sequences per person) for each body part were computed. For each subject only the four body parts identified as most beautiful, most ugly, most satisfying and most dissatisfying were considered for the analyses.

### Statistical Analyses

Normality assumption was true in all three groups for duration and frequency data (Kolmogoroff-Smirnoff p*s* > .09) and nearly all scale and subscale scores except EDE-Q Weight Concern in AN (*p* = .007), BDI in CG (*p* = .05) and EDE-Q Eating Concern in CG (*p* = .003). Multivariate analyses of variance (MANOVA) were computed to analyze group differences in descriptive variables and body part ratings, followed by subsequent univariate analyses of variance (ANOVA) and post-hoc pairwise comparisons if feasible. With eye movement data, separate analyses were undertaken for duration and frequency data. Several 2 (Dimension: beauty vs. satisfaction) x 2 (Body Part: ugly/dissatisfying vs. beautiful/satisfying) x 3 (Group: AN vs. BN vs. CG) repeated measures ANOVAs were conducted. In response to significant effects, subsequent oneway ANOVAs and group-wise paired t-tests (one-tailed, given directed hypotheses based on previous results, [33, 37]) were applied. Controlling for depression, AN and BN were merged and divided in two groups based on the clinical cut-off score for the BDI total score (BDI ≥ 17). In the CG, one subject was omitted for this analysis due to a BDI total score greater than 17. Effects on duration and frequency were analyzed by 2 (Dimension: beauty vs. satisfaction) x 2 (Body Part: ugly/dissatisfying vs. beautiful/satisfying) x 3 (Depression Group Status [D-Group]: BDI high vs. BDI low vs. CG) repeated measures ANOVAs, subsequent oneway ANOVAs and group-wise paired t-tests (one-tailed). A 3 (Group: AN, BN, CG) x 5 (Time: time 1 vs. time 2 vs. time 3 vs. time 4 vs. time 5) repeated measures ANOVA was used to analyze the course of the two SAM subscales *discomfort* and *arousal*. Subsequently, separate ANOVAs and appropriate post-hoc tests for each assessment point were computed.

## Results

### Beauty ratings and satisfaction with body parts

AN, BN and CG did not differ in the indicated most beautiful (*χ* ^*2*^ [14] = 19.39, *p* = .15), most ugly (*χ* ^*2*^ [14] = 19.39, *p* = .15), most satisfying (*χ* ^*2*^ [14] = 19.39, *p* = .15) and most dissatisfying (*χ* ^*2*^ [14] = 19.39, *p* = .15) body parts (see Table 2). Patients and participants in the CG agreed fairly well in the selection of the most ugly and dissatisfying body parts, which were mainly belly, thigh and hip (chosen as most ugly by 56.6% of AN, 81.5% of BN and 87.6% of CG, and chosen as most dissatisfying by 68.8% of AN, 87.5% of BN and 87.6% of CG). In the selection of most beautiful and satisfying body parts, more variation occurred, though mainly body parts located in the upper part of the body, i.e. shoulders, décolleté, breast, arms or stomach were chosen. As can be seen in Table 2, groups differed in ratings of beauty and satisfaction of these body parts (*F* [8,80] = 4.47, *p* < .001, η^2^ = .31) and for each of these body parts (Table 2).

**Table 2:** Selected body parts and individual ratings.

variable	AN (n = 16)	BN (n = 16)	CG (n = 16)	test statistic	*p*	η^2^	Post-hoc Tests
Most beautiful body part (*N*)							
shoulder	3	5	1				
décolleté	0	2	6				
bosom	1	2	3				
arms	2	2	2				
belly	5	3	3				
hip	1	2	0				
thigh / legs	2 / 2	0 / 0	1 / 0				
Individual ratings^1^	3.92 (1.38)	4.50 (0.82)	5.19 (0.75)	F(2, 42) = 5.90	0.006	0.22	AN < CG
Most ugly body part (*N*)							
shoulder	1	1	0				
décolleté	1	1	0				
bosom	2	1	2				
arms	2	0	0				
belly	5	9	4				
hip	2	0	5				
thigh / legs	2 / 1	4 / 0	5 / 0				
Individual ratings^1^	2.00 (1.00)	1.75 (1.13)	3.13 (0.89)	F(2, 42) = 8.30	0.001	0.28	AN, BN < CG
Most satisfying body part (*N*)							
shoulder	2	4	1				
décolleté	1	3	5				
bosom	1	2	3				
arms	2	5	2				
belly	4	1	2				
hip	1	1	0				
thigh / legs	3 / 2	0 / 0	3 / 0				
shoulder							
Individual ratings^1^	3.62 (1.66)	4.94 (1.00)	5.38 (0.72)	F(2, 42) = 8.84	0.001	0.20	AN < BN, CG
Most dissatisfying body part (*N*)							
shoulder	1	0	0				
décolleté	1	1	1				
bosom	1	1	1				
arms	1	0	0				
belly	7	8	6				
hip	2	0	5				
thigh / legs	2 / 1	6 / 0	3 / 0				
Individual ratings^1^	2.23 (1.30)	1.56 (1.09)	2.88 (1.09)	F(2, 42) = 5.17	0.01	0.20	BN < CG

Notes. Standard deviations are given in parentheses. Due to multiple comparisons, statistical significance was set to p = .01. Group differences are based on post-hoc Scheffé-tests, when F values were significant. Only significant differences are denoted.

^1^ = Individuals ratings on a 6-point rating scale (1 = ugly/dissatisfied, 6 = beautiful/satisfied).

### Gaze duration

**Fig 1:**
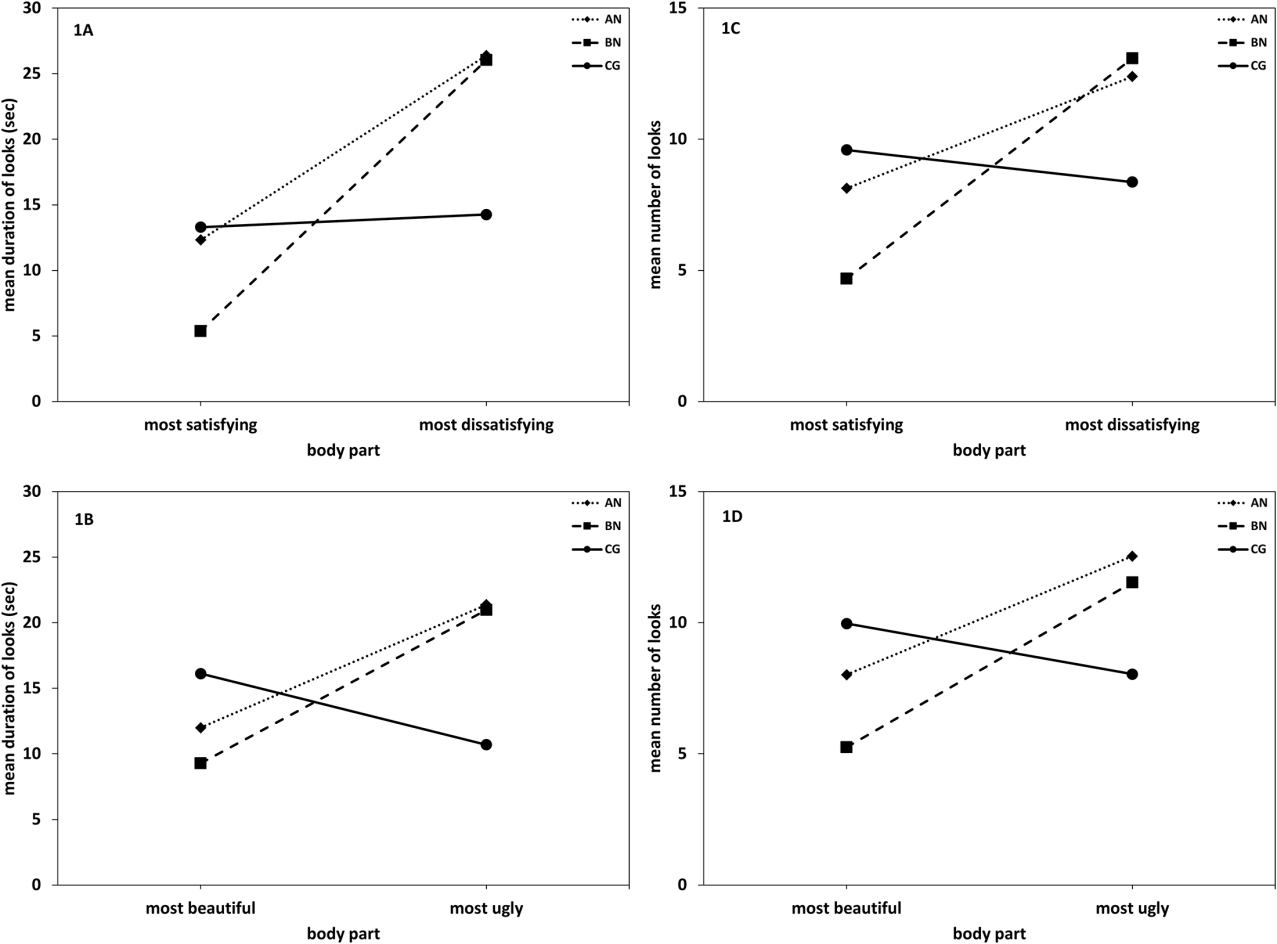
Duration and number of looks towards the most satisfying/most beautiful and most dissatisfying/most ugly body part. Eye movement pattern in patients with anorexia nervosa, bulimia nervosa and in the healthy control group. Duration (in seconds [sec]) of looks at most satisfying/dissatisfying [1A] and beautiful/ugly [1B] body parts and frequency of looks at most satisfying/dissatisfying [1C] and most beautiful/ugly [1D] body parts. AN = anorexia nervosa, BN = bulimia nervosa, CG = healthy control group.

MANOVA results for duration data revealed significant effects of Body Part (*F* [1, 45] = 7.50, *p* = .01, η^2^ = .14), Group × Body Part (*F* [2, 45] = 3.14, *p* = .05, η^2^ = .12) and Dimension × Body Part (*F* [1, 45] = 4.25, *p* = .05, η^2^ = .09). Both AN (*t* [15] = -2.29, *p* = .02, *d* = .57) and BN (t [15] = -2.79, *p* = .005, *d* = .70) spent more time looking at their most dissatisfying than most satisfying body part (Fig 1A). Comparably, both AN (*t* [15] = -1.72, *p* = .05, *d* = .43) and BN (*t* [15] = -1.54, *p* = .05, *d* = .38) patients gazed longer at their most ugly relative to their most beautiful body part (Fig 1B). For the CG (Fig 1A and 1B), significant differences were found neither for the beauty dimension (*t* [15] = 1.18, *p* = .13, *d* = .30) nor for the satisfaction dimension (*t* [15] = -.21, *p* = .42, *d* = .05).

### Gaze frequency

MANOVA resulted in significant effects of Body Part (*F* [1, 45] = 7.66, *p* = .01, η^2^ = .15) and Group × Body Part (*F* [2, 45] = 4.59, *p* = .02, η^2^ = .17). Both BN (*t* [15] = -1.84, *p* = .045, *d* = .86) and AN (*t* [15] = -1.84, *p* = .045, *d* = .46) patients looked more frequently at their most dissatisfying compared to most satisfying body part (Fig 1C). Likewise, both BN (*t* [15] = -2.46, *p* = .015, *d* = .62) and AN (*t* [15] = -1.76, *p* = .05, *d* = .44) patients gazed more frequently at their most ugly compared to their most beautiful body part (Fig 1D). For the CG (Fig 1C and 1D) significant differences were found neither for the beauty dimension (*t* [15] = -.97, *p* = .17, *d* = .24) nor for the satisfaction dimension (*t* [15] = .71, *p* = .24, *d* = .18).

### Correlations of gazes and self-reported body dissatisfaction (BSQ)

There were significant correlations (one-tailed) between body dissatisfaction and gaze frequency towards the most ugly (*r* = .29, *p* = .024), most dissatisfied (*r* = .31, *p* = .017), most beautiful (*r* = —.40, *p* = .002) and most satisfied (*r* = —.47, *p* < .001) body part. Body dissatisfaction also significantly correlated with gaze duration towards the most ugly (*r* = .26, *p* = .040), most beautiful (*r* = —.25, *p* = .085) and most satisfied (*r* = —.31, *p* = .036) body part, but not with gaze duration towards the most dissatisfied (*r* = .21, *p* = .078) body part.

### Influence of depression severity on gaze patterns

Eating disordered patients with high and low BDI (D-Group: BDI-high vs. BDI-low) and the CG were compared (see also statistical analyses section for Group formation). MANOVA on gaze duration revealed a main effect Body Part (*F* [1, 44] = 4.77, *p* = .03, η^2^ = .10), an interaction effect Body Part × D-Group (*F* [2, 44] = 3.21, *p* = .05, η^2^ = .13) and an interaction effect Body Part × Dimension (*F* [1, 44] = 5.99, *p* = .02, η^2^ = .12).

**Fig 2:**
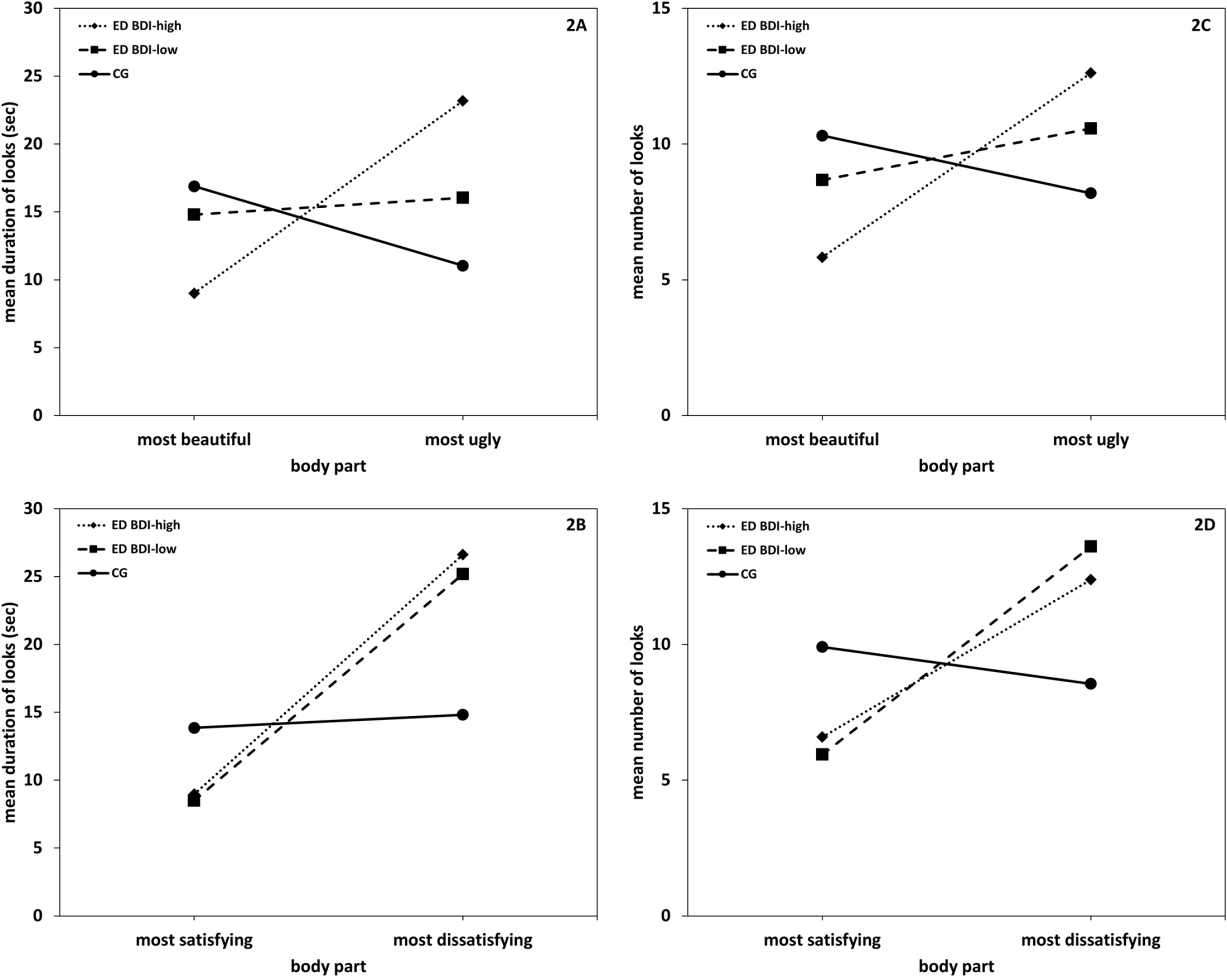
Duration and number of looks towards the most satisfying/most beautiful and most dissatisfying/most ugly body part. Eye movement pattern in the eating disorder groups with high and low severity of depression and the healthy control group. Duration (in seconds) of looks at beautiful/ugly [2A] and most satisfying/dissatisfying [2B] body parts and frequency of looks at most beautiful/ugly [2C] and most satisfying/dissatisfying [2D] body parts. ED = group with eating disorders; BDI-high = Beck Depression Inventory scores with BDI ≥17; BDI-low = BDI < 17, CG = healthy control group.

Eating disorder patients in the BDI-high group looked longer at the most ugly relative to the most beautiful body part (*t* [22] = 2.51, *p* = .01, *d* = .52; Fig 2A) and at the most dissatisfying compared to the most satisfying body part (*t* [22] = 3.05, *p* = .003, *d* = .64; Fig 2B). Likewise, analyses on gaze duration in eating disorder patients in the BDI-low group were significant for satisfaction based analyses (*t* [8] = 1.89, *p* = .05, *d* = .63, Fig 2B), they were however, far from significance with regard to the beauty dimension (*t* [8] = .17, *p* = .44, *d* = .06; 2A).

MANOVA on frequency revealed a similar pattern with a main effect of Body Part (*F* [1, 44] = 5.27, *p* = .03, η^2^ = .11), an interaction effect Body part × Group (*F* [2, 44] = 4.06, *p* = .02, η^2^ = .16) and an interaction effect Dimension × Body Part × Group (*F* [2, 44] = 3.48, *p* = .04, η^2^ = .14). Eating disorder patients in the BDI-high group looked more often at the most ugly relative to the most beautiful body part (*t* [22] = 3.35, *p* < .01, *d* = .70; Fig 2C) and at the most dissatisfying compared to the most satisfying body part (*t* [22] = 2.92, *p* < .01, *d* = .61; Fig 2D). Likewise, gaze analyses on gaze frequency in eating disorder patients in the BDI-low group were significant for satisfaction based analyses (*t* [8] = 2.24, *p* = .03, *d* = .75; Fig 2D), they were however, far from significance with regard to the beauty dimension (*t* [8] = .52, *p* = .31, *d* = .17; Fig 2C).

### Course of mood and arousal during mirror exposure

**Fig 3:**
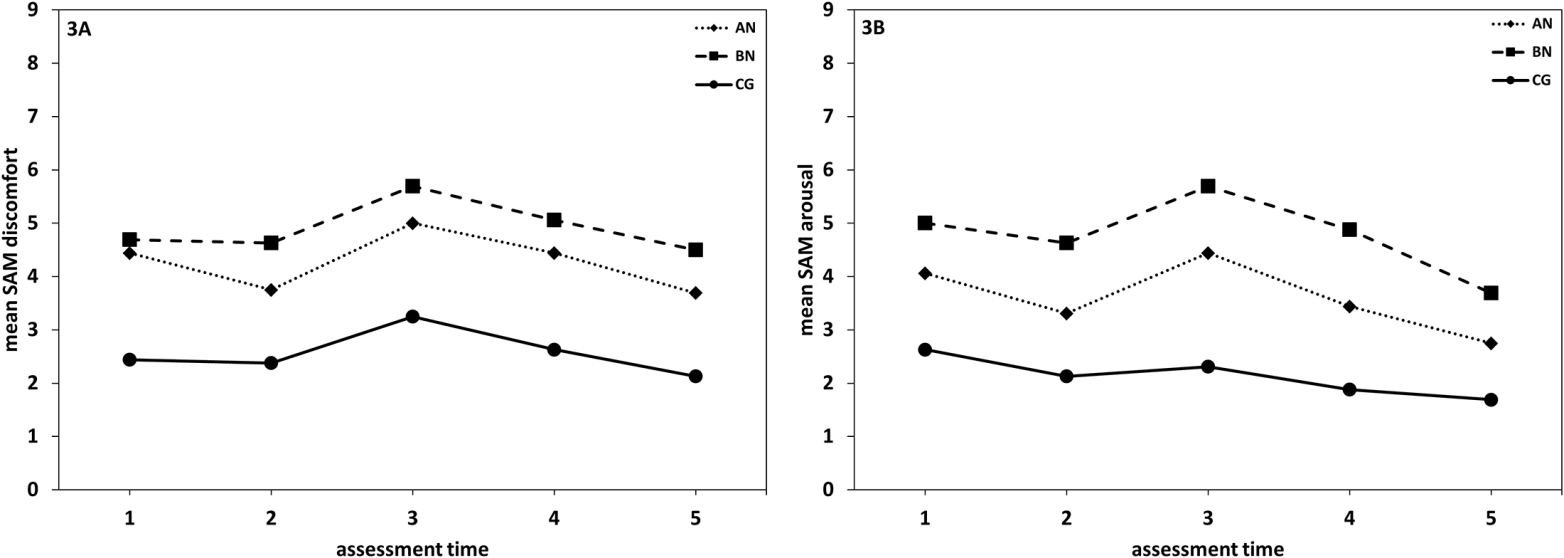
Time course of discomfort and arousal. Time course of SAM discomfort (Fig 3A) and arousal (Fig 3B) in patients with anorexia nervosa (AN), bulimia nervosa (BN) and the healthy control group (CG). Time points: 1 = at beginning, 2 = after relaxation, 3 = after body exposure, 4 = after changing (approx. 10 min after body exposure), 5 = at termination (approx. 35 min after body exposure). Scale: SAM discomfort: 1 = very comfortable, 9 = very uncomfortable; SAM arousal: 1 = lowly aroused, 9 = highly aroused.

For the SAM subscale discomfort (Fig 3A), significant main effects were found for Time (*F* [4, 42] = 6.61, *p* < .001, η^2^ = .13) and Group (*F* [2, 45] = 12.60, *p* < .001, η^2^ = .36). Post-hoc paired comparisons between measurement points revealed that discomfort was highest directly after body exposure (time point three), differing significantly from all other time points (*t*s [47] > 2.48, *p*s ≤ .02, *d*s = .38−.67). Whereas discomfort increased from pre to post-mirror exposure (i.e. from time point 2 to time point 3: *t* [47] = -4.45, *p* < .001, *d* = .64), it decreased thereafter (i.e. time point 3 to time points 4 and 5: *t*s [47] ≥ 2.79, *p*s < .01, *d*s = .41−.67) and returned to baseline level (i.e. time point 1 to time points 4 and 5: *ts* [47] ≤ 1.39, *ps* ≥ .17, *ds* = .09−.20). AN and BN patients experienced comparable levels of discomfort throughout (*F* [1, 30] = 1.33, *p* = .26, η^2^ = .04), but significantly more discomfort than participants in the CG (AN vs. CG: *F* [1, 30] = 14.77, *p* = .001, η^2^ = .33; BN vs. CG: *F* [1, 30] = 29.42, *p* < .001, η^2^ = .50).

For the SAM subscale arousal (Fig 3B), significant main effects were found for Time (*F* [4, 42] = 9.24, *p* < .001, η^2^ = .17) and Group (*F* [2, 45] = 16.90, *p* < .001, η^2^ = .43). AN and BN patients experienced a significantly higher arousal than participants in the CG (AN: *F* [1, 30] = 11.72, *p* = .002, η^2^ = .28; BN: *F* [1, 30] = 44.01, *p* < .001, η^2^ = .60). In addition, BN patients self-reported a higher arousal than AN patients (*F* [1, 30] = 4.92, *p* = .03, η^2^ = .14). Post-hoc comparisons between measurement points revealed a significant increase in arousal from pre to post mirror exposure (i.e. time point two to time point three: *t* [47] = 2.87, *p* < .01, *d* = .41) and a significant decrease thereafter (i.e. time point three to time point four to time point five: *t*s [47] > 4.06, *p*s < .001, *d* = .58−.63). Thereby, arousal was significantly lower at the end of the procedure than at the beginning (i.e. time point one to time point five: *t* [47] = 3.88, *p* < .001, *d* = .56).

## Discussion

The aim of the present study was to test whether women with AN and BN compared to women without eating disorders are characterized by an attentional bias towards the body part they are most dissatisfied with or find most ugly compared to the body part they are most satisfied with or find most beautiful. We further tested for differences between AN and BN in the attention allocation towards these body parts.

In line with our hypotheses, both women with AN and BN spent more time looking and gazed more often at their most dissatisfying than to their most satisfying body part. Likewise, they gazed longer and more often at their most ugly relative to their most beautiful body part. Contrasting this result pattern, women in the CG displayed an even distribution of eye gazes on these body parts both with regard to gaze duration and gaze frequency. From a theoretical perspective [58, 59], the increased attentional bias for the most negatively valenced body part may lead to a persistence and aggravation of eating disordered patients’ negative body image, as qualifying additional information or neutral and positive body information are not attended to and are thus neglected. Along with this and with the exception of gaze duration towards the most dissatisfied body part, correlation analyses revealed that the stronger the attentional bias towards the most negatively valenced body part, the more dissatisfied participants were with their body. Conversely, the stronger the attentional bias towards the most positively valenced body part, the more satisfied participants were with their body.

Our results are in line with other studies that emphasize body dissatisfied and eating disordered women to be characterized by a hypervigilance towards problematic body zones [23, 28, 32, 33, 35, 37]. They however contrast studies that found body dissatisfied and eating disordered women [24, 25, 36] to avoid negatively evaluated body parts. At the behavioral level, both body checking and body avoidance are highly fluctuating features of AN and BN [60]. Thus, sample differences with regard to body avoidance and body checking across studies might have contributed to the contradicting results. Notably, by negative reinforcement both body checking and avoidance are thought to reduce immediate fear of weight gain and accompanying negative affect [59]. In the long-run, though, both behaviors are thought to contribute to the maintenance of AN and BN, as on the one hand an extreme avoidance of certain body areas does not enable to disconfirm pathological body-related cognitions, and repeated checking on problematic areas—on the other hand–increase negative self-evaluation [61, 62]. To better understand the reported differential gaze patterns and their role in the maintenance of eating disorders, future studies should control for body checking and avoidance at least at the self-report level.

Beyond this argument, though, other reasons might account for the hypervigilance found in AN and BN patients in the present study. As gaze patterns not automatically reflect attention allocation, we used a thought sampling technique during the recording of eye movements in order to increase data validity. By instructing participants to look at the mirror and speak aloud their thoughts and emotions, though, we might have reduced naturally occurring avoidance behavior. Future studies could better control for such a possible effect by manipulation of instructions or assessment methods (e.g., with and without thought sampling technique).

Contrary to our hypothesis, BN participants’ attentional bias towards negatively valenced body parts was not increased compared to the AN group. When presenting self- and other body pictures concurrently, AN and BN patients were found to display different attentional patterns [30]. Thus, differences between AN and BN might be evident at the level of social comparison, but not at the level of attentional distribution towards body areas within their own body. Notably, the differential hypothesis for AN and BN patients was postulated on the basis of previous studies showing that women with BN self-report higher levels of cognitive-affective body image disturbances compared to women with AN [6]. In the present study, though, BSQ and EDE-Q scores were comparable across eating disorder diagnoses, possibly because all our eating disorder participants were inpatients. In fact, according to the German Guidelines for the treatment of eating disorders [63], individuals with BN should only be treated in inpatient settings when outpatient treatment failed or in case of high comorbidity or a high level of symptomatology. Thus, symptomatology in our BN participants might have been particularly severe.

Given that Major Depression is a frequent comorbid disorder of AN and BN [64], and that negative mood has been shown to increase both body size perception [65] and the selective attention for disorder-relevant stimuli [66], subsequent analyses were conducted in order to control for the effects of depression severity on the attentional distribution towards the areas of interest during mirror exposure. To this end, AN and BN patients were merged and divided into a group with high (BDI-high group) and low (BDI-low group) depressive symptomatology. Comparisons with the non-depressed control group revealed that the BDI-high group gazed significantly longer as well as more often both at the most ugly and most dissatisfying relative to the most beautiful and most satisfying body part. By contrast, a weaker pattern was found in the BDI-low group. As such, relative to the non-depressed control group, eating disordered participants in the BDI-low group gazed longer and more frequently at the most dissatisfying relative to their most satisfying body part. There were, however, no gaze-related between-group differences on the beauty dimension. This strengthens the assumption that perception of beauty and satisfaction of the self-body may be different constructs, which are more or less affected by current mood. It also emphasizes that differential result patterns found in previous studies might be attributable to the current state of mood and the outcome variable (beauty vs. satisfaction vs. attractiveness dimension). Furthermore, to better understand the causal role of mood in the attentional processing of the self-body, future studies should experimentally test the effects of mood on the attention allocation towards the most beautiful/ugly and the most satisfying/dissatisfying body part, e.g., in a within-design.

Contrary to our hypotheses, patients with AN and BN did not experience more discomfort and arousal during the mirror exposure than participants in the CG. Instead, all participants self-reported an increase of discomfort and arousal from pre to post mirror exposure, which returned to baseline level by the end of the experiment. These results contradict previous studies [39, 40, 67] reporting a stronger increase in negative affect in patients with eating disorders compared to controls during the course of a mirror exposure. On the other hand, other studies reported a significant increase in distress during mirror exposure also in non-eating disordered controls [68]. First of all, the different measures across the various studies might tap different underlying constructs. As such some studies assessed a range of negative emotions during the course of mirror exposure [40, 67], while others assessed subjective units of distress [68]. Furthermore, as considerable time elapsed from the beginning of the experiment to the mirror exposure (baseline measures, setting up the eye tracking device, calibration), anticipatory anxiety might have led to the overall increased discomfort and anxiety in the AN and BN group. Notably, patients with BN self-reported a significantly higher arousal than AN patients. This converges with previous results [6], which showed that BN patients are characterized by higher levels of cognitive-affective body image disturbances. Nevertheless, higher arousal levels did not—against our hypothesis- lead to a higher attentional processing of the most ugly/most dissatisfied body part. As arousal was not assessed in an experimental control condition, it is unclear whether BN patients were overall more anxious or were more anxious given the (upcoming and ongoing) mirror exposure. Nevertheless, given the somewhat weaker hypothesized gaze pattern results in the low BDI-low group it would be important for future studies to experimentally test the effects of arousal and negative emotions on the attentional processing of the self-body.

Several other limitations have to be noted. First of all, all eating disordered patients were inpatients, thus, the results may not be representative for untreated AN and BN patients or eating disorder patients in the outpatient setting. Second, several AN and BN patients dropped out of the study after being explained the experiment. Being inpatients, they might have felt overwhelmed with the study procedure. As the ethical protocol highlighted that participants were allowed to drop out without reason, we have no information on why these participants did not show up to the scheduled eye tracking appointment. Furthermore, a considerable number of participants had to be excluded due to technical problems and missing data. Even though there were no significant differences between participating patients and patients that had withdrawn or had been excluded due to technical problems with regard to demographic variables, overall and eating pathology, it cannot be excluded, that they might have differed with regard to the attentional processing of the self-body. Third, no control condition was included. As such, it remains unclear whether the “negativity bias” found in AN and BN pertains to the self-body, or, whether these patients are characterized by a general negativity bias for body-related information or even body-unrelated (negative) information. Beyond that, a less naturalistic, but stronger experimental approach would have enabled to better test for the time course of the attentional processing of the self body in AN and BN. This is especially important for future studies, as—in subclinical samples—both automatic and strategic processes were found to influence the attentional processing of body-related cues [26, 27]. This notwithstanding, in comparison to photos, mirror exposure can be considered an ecologically more valid procedure, thereby yielding stronger treatment implications.

Finally, another limitation of this correlational study is that causal effects are difficult to determine. One way to test for causal effects of body-related attentional biases in AN and BN would be to manipulate the latter and to test for possible effects in (more) meaningful variables. Notably, experimental evidence in the non-clinical domain suggests that the selective attentional bias for unattractive body parts is causally linked to body dissatisfaction [69, 70]. Of more clinical relevance, it has also been demonstrated that a retraining of the attention towards self-defined attractive body parts increases body satisfaction in body dissatisfied women [71]. However the latter was shown to be more difficult, needing a more extensive retraining [71]. In line with this, future studies could test whether the bias towards negatively valenced body parts in AN and BN is modifiable by, e.g., repeated mirror exposure or attention bias modification, and whether its reduction leads to an improvement in body satisfaction and thus an improvement in disorder-relevant variables such as weight gain and normalization of food consumption.

Taken together, the present study yielded evidence of an increased attentional processing of negatively valenced body parts during an unguided mirror exposure in patients with AN and BN compared to HC. Especially eating disordered patients with high self-reported severity of depression are characterized by longer and more frequent gazes towards negatively compared to positively valenced body parts.
